# Supplementary figures and images for: Supplementation with Red Wine Extract Increases Insulin Sensitivity and Peripheral Blood Mononuclear Sirt1 Expression in Nondiabetic Humans
Source: Nutrients. 2020 Oct 12;12(10):3108. doi: 10.3390/nu12103108 (PMC7600896; doi:10.3390/nu12103108)

## Supplemental Figure

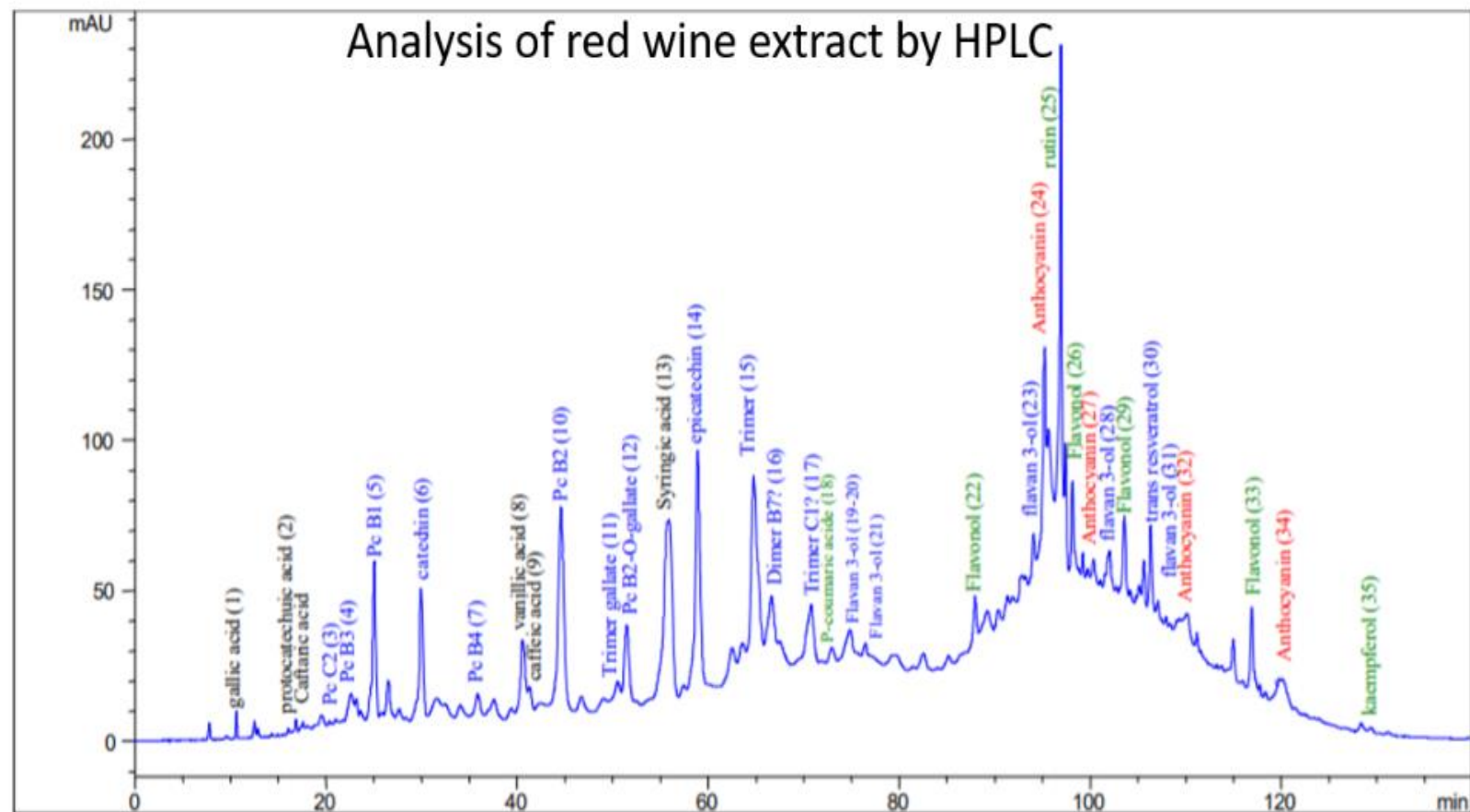

Supplement: Supplementary file 1 [file nutrients-12-03108-s001.pdf]
